# Molecular fingerprinting of complex grass allergoids: size assessments reveal new insights in epitope repertoires and functional capacities

**DOI:** 10.1186/s40413-017-0146-3

**Published:** 2017-04-24

**Authors:** S. Starchenka, A. J. Bell, J. Mwange, M. A. Skinner, M. D. Heath

**Affiliations:** Allergy Therapeutics, Ltd, Dominion Way, Worthing, BN14 8SA UK

**Keywords:** Immunotherapy, Allergen, Complex grass, Lol p 1, Allergoid, Epitope, Immunogenicity, Allergenicity

## Abstract

**Background:**

Subcutaneous allergen immunotherapy (SCIT) is a well-documented treatment for allergic disease which involves injections of native allergen or modified (allergoid) extracts. The use of allergoid vaccines is a growing sector of the allergy immunotherapy market, associated with shorter-course therapy. The aim of this study was the structural and immunological characterisation of group 1 (Lol p 1) IgG-binding epitopes within a complex mix grass allergoid formulation containing rye grass.

**Methods:**

HP-SEC was used to resolve a mix grass allergoid preparation of high molecular weight into several distinct fractions with defined molecular weight and elution profiles. Allergen verification of the HP-SEC allergoid fractions was confirmed by mass spectrometry analysis. IgE and IgG immunoreactivity of the allergoid preparations was explored and Lol p 1 specific IgG-binding epitopes mapped by SPOT synthesis technology (PepSpot™) with structural analysis based on a Lol p 1 homology model.

**Results:**

Grass specific IgE reactivity of the mix grass modified extract (allergoid) was diminished in comparison with the mix grass native extract. A difference in IgG profiles was observed between an intact mix grass allergoid preparation and HP-SEC allergoid fractions, which indicated enhancement of accessible reactive IgG epitopes across size distribution profiles of the mix grass allergoid formulation. Detailed analysis of the epitope specificity showed retention of six Lol p 1 IgG-binding epitopes in the mix grass modified extract.

**Conclusion:**

The structural and immunological changes which take place following the grass allergen modification process was further unravelled revealing distinct IgG immunological profiles. All epitopes were mapped on the solvent exposed area of Lol p 1 homology model accessible for IgG binding. One of the epitopes was identified as an ‘immunodominant’ Lol p 1 IgG-binding epitope (62-IFKDGRGCGSCFEIK-76) and classified as a novel epitope. The results from this study support the concept that modification allows shorter-course therapy options as a result of providing an IgG epitope repertoire important for efficacy. Additionally, the work paves the way to help further develop methods for standardising allergoid platforms.

**Electronic supplementary material:**

The online version of this article (doi:10.1186/s40413-017-0146-3) contains supplementary material, which is available to authorized users.

## Background

Allergy presents an exaggerated immune response towards foreign allergens due to impaired immune tolerance. Type I hypersensitivity affects 20% of the world population and has clinical symptoms of asthma, conjunctivitis and anaphylaxis [1]. In healthy individuals, allergic response to the various environmental allergens is suppressed by a combination of various innate and adaptive immune mechanisms which maintain immune tolerance. This mechanism of immune tolerance is impaired in allergic individuals. IgE activated mast cells contribute to biased Th2 and Th17 responses by supressing and re-programming T regulatory cells [2].

In recent years, several approaches have been further developed to improve the efficacy and safety profile of specific immunotherapy. It includes synthesis of hypoallergenic recombinant proteins [3], immunogenic peptides [4], or using modified protein extracts with reduced allergenicity combined with novel Th1 second generation adjuvants, such as Monophosphoryl Lipid A (TLR-4 agonist) [4, 5]. Introduction of novel adjuvants in combination with chemically modified allergens (allergoids) [6] has been demonstrated to reduce side effects of specific immunotherapy, increase clinical efficacy of allergy treatment and reduce the number of injections [7–9].

The design of commercial allergoid vaccines is based on chemical modifications of native allergen molecules by potassium cyanate, formaldehyde or glutaraldehyde. The chemicals such as gultaraldehyde, react with specific amino acid side chains from protein structures resulting in conformational changes in integral protein structure/function. Glutaraldehyde, for example, enables intramolecular and intermolecular cross-linking and formation of polymer molecules with large molecular mass [10–12]. Modification of allergens generally results in more limited capacity of IgE binding that is dependent on the conformation of a native protein. The extensive cross-linking of proteins results in the disruption of these predominantly conformational IgE epitopes. On the contrary, IgG binding is largely based on sequential amino acid epitopes, therefore while the modification of allergoids diminishes allergenicity, their immunogenicity is thought to be preserved. As a consequence, it presents a unique treatment option since it is feasible to deliver a higher dose of the active substance (allergoid) without compromising safety [8, 9, 11, 13].

Literature sources report that allergoids harbor their respective relevant allergens, which are present in native extracts. It was demonstrated for birch pollen [10], house dust mites [14], parietaria pollen [15] and timothy grass pollen [12]. However, there is limited data available regarding allergen content for complex allergoid vaccines containing mixtures of cross-reactive species. The large allergoid size/structure complicates their characterisation using traditional techniques, which are commonly used for native allergen extract characterisation. Native and modified extracts are not are not two discrete preparations but are a more complex heterogeneous formula of native and modified allergen sizes and structures, respectively. According to the Paul Ehrlich Institute, recommendations on the standardization and regulation of allergen products characterisation of allergoid formulations should be done, for example, using mass spectrometry and size exclusion chromatography [16]. Some studies reported the size of polymerised allergoids to cover a 1000–3000 kDa molecular weight range, others in the range 200–20000 kDa. Carnes et al. (2008) studied allergoids of *Betula alba* and found that 60% of polymerised molecules had a molecular mass in the range of 1000–3000 kDa, 15% of the molecules had molecular weight of 3000–10000 kDa and 9% exceeded 10000 kDa.

Clinical studies of a complex grass allergoid vaccine – Pollinex Quattro™ (Allergy Therapeutics Plc) - report modulation of non-specific and specific immune response during an ultra-short therapy course and, therefore, successful desensitisation of treated subjects [17]. Grass pollen is the most common allergen encountered worldwide [18, 19]. Around 40% of allergic individuals are sensitized against pollen allergens [20]. Rye grass (*Lolium perenne*) is a major allergenic source in Europe, North America and Australia [1, 21]. Aqueous extract of this grass contains 17 characterised allergenic proteins with molecular weights ranging between 11 kDa and 89 kDa. 95% of grass sensitized individuals show IgE reactivity against group 1 rye grass allergens [1, 22, 23]. Homologous group 1 grass allergens are glycoproteins from the expansins family with a molecular weight of 30–35 kDa [24].

Epitopes are specific regions of the allergen, which are recognized by the components of the immune system. There are two main types of epitopes: discontinuous (conformational) and continuous (sequential). Conformational (e.g. IgE) epitopes are influenced by protein folding and often have key residues derived from the different parts of the linear sequence. Sequential (i.e. IgG) epitopes are not dependant on the three-dimensional structure and are usually represented by successive residues from the linear sequence. B cells recognize conformational and linear epitopes of allergens via interaction of the transmembrane antibody receptors [25–27].

We report the structural and functional characterisation of a complex grass mix allergoid formulation, including the antibody binding epitopes for the group 1 allergen, Lol p 1. Lol p 1 IgG binding epitopes were identified using synthetic overlapping peptides. ELISA inhibition platform was used to determine the presence of major and minor Lol p 1 IgG binding epitope sequences in the complex mix grass allergoid formulation and HP-SEC fractions derived from it.

## Methods

### Mix grass pollen native and modified extracts

Grass pollens were purchased from Pharmallerga (Czech Republic) and Allergon (Sweden) and consisted of a 5% w/v extract mix of the following homologous species: bent grass (*Agrostis capillaris*), foxtail meadow (*Alopecurus pratensis*), sweet vernal (*Anthoxanthum odoratum*), false oat (*Arrhenatherum elatius*), brome (*Bromus inermis*), dogstail crested (*Cynosurus cristatus*), cocksfoot (*Dactylis glomerata*), fescue meadow (*Festuca pratense*), yorkshire fog (*Holcus lanatus*), rye grass (*Lolium perenne*), timothy grass (*Phleum pratense*), meadow grass (*Poa pratensis*). A 5% w/v grass extract was prepared in a sodium phosphate solution (pH 6.5) by extracting on auto-titration (using (NaOH / HCl)) for 24 h at 2 to 8 °C at pH 7.0. The extract was clarified using coarse filtration with Whatman 54 paper filter (Millipore, UK) followed by fine filtration using disc filters 8.0 μm-0.45 μm (Millipore, UK). The grass extract was further dialysed against to remove low molecular weight impurities. Following diafiltration, the extract was sterile filtered with 0.22 μm filters (Millipore, UK). The 5% w/v mix grass extract was modified with aqueous 0.25% v/v glutaraldehyde (Fisher Scientific Ltd., UK) for 2 h continuously stirring at room temperature. The modified extract was diafiltered using Millipore Pellican 2 ultrafiltration cassette (Millipore, UK). Native and modified extracts were stored at 2 to 8 °C.

### HP-SEC of the mix grass modified extract

The mix grass allergoid fractions were collected as the mobile phase eluates from the SEC column at distinct time intervals. HP-SEC was performed on an Agilent 1100 HPLC system with a UV detector (280 nm). Allergoid fractions with molecular weight distribution 113,736 Da – 2,249,594 Da were separated using a 7.8 × 300 mm BioSep-SEC-S3000 column (Phenomenex, UK). The column was equilibrated with 0.1 M Sodium phosphate buffer (pH 6.8) (13.8 g NaH_2_PO_4_·H_2_O (Fisher Scientific Ltd., UK), 14.2 g Na_2_HPO_4_ (Fisher Scientific Ltd., UK), 2 L of HPLC grade water (Fisher Scientific Ltd., UK)) at flow rate 0.5 ml/min and ambient temperature at least 3 h prior the analysis. An isocratic mobile phase of 0.1 M Sodium phosphate buffer (pH 6.8) at a flow rate of 0.5 ml/min at ambient temperature was used for column calibration and separation of the fractions.

HP-SEC system performance was checked using an aqueous SEC1 column check standard protein mix ALO-3042 (Phenomenex, UK). Protein peak resolution (R_s_) was calculated for the IgG standard (Mw 150,000 Da) and Ovalbumin (Mw 44,000 Da) peaks:

$$ {\mathrm{R}}_{\mathrm{s}}=\left({\mathrm{T}}_{\mathrm{R}2}-{\mathrm{T}}_{\mathrm{R}1}\right)/\left({\mathrm{T}}_{\mathrm{W}1}-{\mathrm{T}}_{\mathrm{W}2}\right) $$

Where, T_R1_ – retention time of IgG, T_R2_ - retention time of Ovalbumin, T_W1_ – peak width for IgG, T_W2_ – peak width for Ovalbumin resolution. Acceptance calibration criteria was set up at R_s_ ≥ 13.

Molecular weights of the allergoid fractions were calculated by comparison of retention times (RT) against known molecular weight standards. The standard calibration curve included 6 standards with a molecular weight range of 2,000,000 Da – 66,000 Da and RT 10.3-17.9 min. A gel filtration marker kit MWGF1000 (Sigma) was used to construct the standard calibration curve. Logarithms of the Mw of the standards was plotted against RT of the standards in minutes with an acceptance criteria of R^2^ ≥ 0.95 for the correlation coefficient and Mw ≥ 250,000 for a RT of 15 min. Data analysis was performed using Agilent ChemStation software version B.04.03.

### LC-MS/MS analysis

Mobile phase eluates of each mix grass allergoid fraction collected from HP-SEC were sent to the Advanced Proteomics Facility at the Department of Chemistry, Biochemistry and The Dunn School at the University of Oxford for mass spectrometry analysis. The digested and eluted peptides were analysed on an Orbitrap LTQ mass spectrometer (Thermo Scientific) fitted with a nanospray ion source and using stainless steel nano-bore emitters (both Proxeon Biosystems, Odense, Denmark). Tandem mass spectra were collected in a data-dependent fashion by collecting one full MS scan (*m/z* range: 375–1800) followed by MS/MS spectra of the 5 most abundant precursor ions (in ion trap), both in the Orbitrap detector. The MS/MS data sets were analysed using the Mascot search engine against an annotated UniProtKB/Swiss-Prot databases. The searches were set up with specified constraints in place to reduce the number of random matches.

### Patient sera

Grass specific pool of human IgE sera was acquired from PlasmaLab International (Everett, WA, USA). Grass specific sera was screened and collected from 10-15 atopic donors sensitized to *Pooideae* subfamily of the grasses and clinically diagnosed as allergic to grass species. Sensitization profile was verified via quantification of specific IgE by RAST, scores were determined according to the manufacturer’s technical specification (Thermo Fisher ImmunoCAP^®^ FEIA). The study was approved by ethics committee and informed consent was obtained before the commencement of the study.

### In vivo immunization (Polyclonal IgG Sera)

For the generation of anti-Lol p 1 immunodominant polyclonal IgG sera, two New Zealand white rabbits were immunized with *Lolium perenne* (rye grass) freeze dried (Lol p 1) material eluted from HP-SEC fractions. Protein content of the immunogen was determined by Bradford protein assay and major Lol p 1 allergen content was confirmed by LC-MS/MS analysis. Rabbits were immunized with 3 subcutaneous injections of the immunogen on the day 0 (0.212 mg of Lol p 1 immunogen), 7 (0.182 mg of Lol p 1 immunogen) and 35 (0.0974 mg of Lol p 1 immunogen). Sample bleeds to assess specific IgG titre were performed on the day 42. Terminal blood samples were collected on the day 49. The study was conducted at Charles River Laboratories International, Inc. Preclinical Services and followed ethical requirements for animal experimentation.

### SDS-PAGE and Western blotting

Each HP-SEC fraction and native grass extract were mixed with sample loading buffer (10 ml HPLC grade water (Fisher Scientific Ltd., UK), 2.5 ml 10 × Tris/Glycine/SDS buffer (Bio-Rad, UK), 3.75 ml glycerol (Fisher Scientific Ltd., UK), 0.475 g SDS (Fisher Scientific Ltd., UK)) containing 20 mg of 1,4–dithiothreitol (DTT) (Fisher Scientific Ltd., UK). Volumes of sample loading buffer were adjusted according to the protein content of each fraction to ensure equal loading. Samples were denatured at 95 °C for 5 min.

Proteins were resolved on a 10-20% Tris–HCl Criterion gel (Bio-Rad, UK) using 10× Tris/Glycine/SDS buffer (Bio-Rad, UK) at 200 V for 1 h. In-gel staining was performed using Pierce® Silver staining kit (Fisher Scientific Ltd., UK) and following the manufacture instructions.

Western blotting was performed by protein transfer onto polyvinyldifluoride membrane using regular iBlot^TM^ (Invitrogen, UK). The blot was blocked with 10% milk diluent (KPL, UK) in DPBS (pH 7.4) for an hour at room temperature and washed with DBPS + 0.3% Tween 20 (Sigma). The blot was incubated overnight at 4 °C with immunoprecipitated anti-Lol p 1 immunodominant polyclonal IgG rabbit sera (1:10,000) (Charles River Laboratories, UK). The membrane was washed with several changes of DBPS + 0.3% Tween 20 (Sigma) and probed with secondary goat anti-rabbit IgG HRP conjugated antibodies (1:20,000) (KPL, UK) for an hour at room temperature. Following the final wash signal was developed using 1-component TMB substrate (KPL, UK). The blot was rinsed with HPLC grade water (Fisher Scientific Ltd., UK) to stop the colour development.

### Immunoreactivity of grass specific IgE human sera against native and modified grass extracts

Ability of grass specific IgE to bind to IgE epitopes in native or modified grass extract was determined using direct ELISA platform. 96-wells microplates (Nunc Maxisorp, Rosklide, Denmark) were coated with native or modified grass extract at a concentration of 20 μg/ml. The plates were incubated overnight at 2 to 8 °C and washed the next morning with DBPS + 0.1% Tween 20. The plates were blocked with 1% BSA for 1 h at room temperature. Grass-specific human IgE sera was applied to the plates at the titre 1 to 1:1024. The plates were incubated for two hours at room temperature. Excess of sera was washed with DBPS + 0.1% Tween. The plates were incubated with secondary goat anti-human IgE HRP conjugated antibody (1:5,000) (KPL, UK) for two hours at room temperature. Excess of secondary antibody was washed with DBPS + 0.1% Tween 20 and signal developed using TMB substrate (KPL, UK). The reaction was stopped with 1.0 orthophosphoric acid (H_3_PO_4_) (Fisher Scientific Ltd., UK) and measured at 450 nm.

### Immunoreactivity of grass specific IgG rabbit sera against native and modified grass extracts

Ability of Lol p 1 specific IgG to bind to IgG epitopes in native or modified grass extract and fractions was determined using direct ELISA platform, as detailed above. HP-SEC allergoid fractions were coasted using 6–15 μg/ml depending on fraction protein concentration. Anti-Lol p 1 immunodominant polyclonal IgG rabbit sera (Charles River Laboratories, UK) was applied to the plates at the titre 1:250 to 1:256 × 10^3^ was diluted on the plates 1 in 2 and the plates were incubated for two hours at room temperature. Excess sera was washed with DBPS + 0.1% Tween 20 (Sigma). The plates were incubated with secondary goat anti-rabbit IgG HRP conjugated antibody (1:5,000) (KPL, UK) for two hours at room temperature. Excess of secondary antibody was washed with DBPS + 0.1% Tween 20 (Sigma) and signal developed using TMB substrate (KPL, UK). The reaction was stopped with 1.0 orthophosphoric acid (H_3_PO_4_) (Fisher Scientific Ltd., UK) and read at 450 nm.

### Lol p 1 epitope screening using PepSpot peptide membrane

The amino acid sequence of Lol p 1 was verified using the UniProtKB (Lol p 1 in UniProtKB/Swiss-Prot) (P14946). SPOT synthesis technology (JPT Peptide Technologies, Germany) was used to bind covalently via C-terminus 63 overlapping synthetic peptides containing 15 amino acids to a Whatman 50 cellulose support (Whatman, UK). Peptides were overlapping by 11 amino acids and acetylated at the N-terminus to prevent degradation.

The membrane was rinsed with HPLC grade methanol (Fisher Scientific Ltd., UK) for 5 min and washed with TRIS buffered saline (TBS) + 0.05% Tween 20 (Sigma) three times. It was followed by blocking with 2% non-fat dry milk concentrate in borate buffer (KPL, UK) diluted with TBS 1/10 for 2 h at room temperature. The wash step was repeated 5 × 5 min to remove excess of blocking buffer. After washing the membrane was incubated with anti-Lol p 1 immunodominant polyclonal IgG sera (1:5,000) (Charles River Laboratories, UK) overnight at 4 °C. On the following day the membrane was washed with TBS + 0.05% Tween 20 (Sigma) five times and probed with the secondary antibody for two hours at room temperature: Goat-anti-Rabbit IgG-HRP (1:20,000) (KPL, UK). The membrane was developed using Prime ECL detection reagent (GE Healthcare, UK) and Amersham ECL Hyperfilm (GE Healthcare, UK).

### Lol p 1 antigenic mapping in the mix grass modified extract

To determine retention of Lol p 1 IgG binding epitopes in the grass modified preparation, 9 synthetic peptides corresponding to Lol p 1 IgG binding sequence identified from a PepSpot screening experiments were synthesized by JPT Peptide Technologies, Germany. Purity of the peptides was determined by HPLC analysis using C18 RP-HPLC column 3 μm 20 × 2 mm and detection at 220 nm. Linear gradient 5-95%B and flow rate 1 ml/min was used with eluent A: 100% H_2_O + 0.05% TFA and eluent B: 100% CH_3_CN + 0.05% TFA. Purity >70% was reported for each peptide. In addition ESI-MS was employed to determine molecular masses of synthesized peptides.

Plate binding efficiency for each of the Lol p 1 specific peptide was verified using direct ELISA. All nine Lol p 1 synthetic peptides exhibited efficient binding capacity to the plate in comparison with a non-specific LSVLDKIYTSPLC control peptide. Absorbance value (>1 AU) for the Lol p 1 specific peptides (above the baseline control of a non-specific peptide) confirmed their validity to be used in ELISA format experiments as a plate-bound antigen. It also confirmed the ability of synthetic peptides directly bind anti-Lol p 1 immunodominant polyclonal rabbit IgG sera.

Mapping of Lol p 1 IgG-binding epitopes was done in the intact mix grass modified extract (allergoid) and in four separate HP-SEC fractions of the allergoid (size range 2,249,594 Da - 113,736 Da). A competitive ELISA platform was used to determine the presence of Lol p 1 epitopes in the allergoid and HP-SEC fractions. Intact allergoid formulation and HP-SEC fractions were used as the soluble inhibitors of anti-Lol p 1 IgG binding to plate bound Lol p 1 synthetic peptides. 96-wells microplates (Nunc Maxisorp, Rosklide, Denmark) were coated with synthetic peptides at concentrations 20 μg/ml (for intact allergoid preparation) and 10 μg/ml (for HP-SEC fractions). The plates were incubated overnight at 2 to 8 °C and washed the next morning with DBPS + 0.1% Tween 20 (Sigma). The plates were blocked with 1% BSA (Sigma) in coating buffer for an hour at room temperature. The wash step was repeated. Inhibition of binding of anti-Lol p 1 immunodominant polyclonal IgG rabbit sera to the plate bound synthetic peptides was performed by applying serial dilutions of soluble phase allergoid inhibitor or HP-SEC fraction inhibitors. 50 μl of anti-Lol p 1 immunodominant polyclonal IgG sera (1:500) (Charles River Laboratories, UK)) were aliquoted in each well except the negative controls. The plates were incubated for two hours at room temperature and washed with DBPS + 0.1% Tween 20 (Sigma) five times. The plates were incubated with secondary goat anti-rabbit IgG HRP conjugated antibodies (1:5,000) (KPL, UK) for two hours at room temperature. Excess of secondary antibodies was washed with DBPS + 0.1% Tween 20 (Sigma) and signal developed using TMB substrate (KPL, UK). The reaction was stopped with 1.0 orthophosphoric acid (H_3_PO_4_) (Fisher Scientific Ltd., UK) and read at 450 nm. Inhibition of IgG binding to the plate-bound peptides was determined using the following equation:

$$ \%\ \mathrm{inhibition} = \left[\left({\mathrm{A}}_0-{\mathrm{A}}_i\right)/{\mathrm{A}}_0\right] \times 100 $$

Where A_0_ - absorbance without the inhibitor and Ai - absorbance with the inhibitor (allergoid or HP-SEC fractions).

### Homology modelling of Lol p 1

A three-dimensional homology model of the Lol p 1 structure was constructed using the Swiss Prot server. A template structure of Phl p 1 with 89.58% sequence identity to Lol p 1 sequence was used to generate a homology model of Lol p 1. Structure was visualised using Chimera version 1.10.

## Results

### Molecular weight distribution and allergen verification of the mix grass allergoid formulation

HP-SEC was used to resolve allergoids of high molecular weight into several distinct fractions with defined molecular weight (Mw) and elution profiles. Mw of the allergoid fractions was determined using a standard calibration curve. A chromatogram profile across a retention time (RT) between 9.0 min and 22.0 min, corresponding to the complex allergoid formulation is provided in (Additional file 1: Figure S1). Four fractions were collected with the size distribution between 2,249,594 Da and 113,736 Da (Table 1).

**Table 1:** HP-SEC fractions from the mix grass allergoid formulation

Collected fractions	Retention time (min)	Mw (Da)
1	10.34	2,249,594
2	12.29	973,404
3	14.21	424,493
4	17.22	113,736

**Fig. 1:**
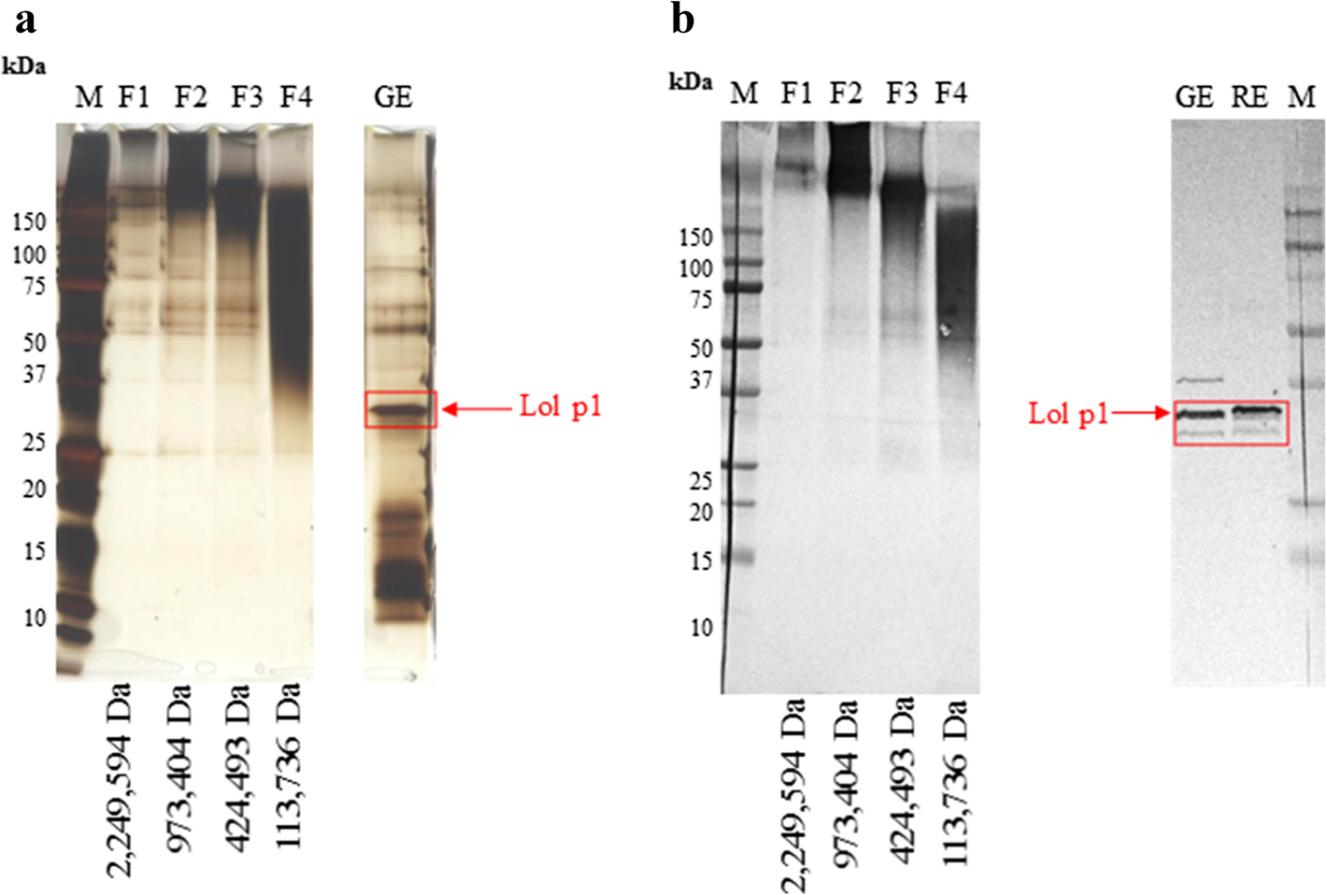
Protein profile and immunoblot of the native mix grass and rye grass extracts vs HP-SEC allergoid fractions preparations. Protein profile (**a**) and immunoblot (**b**) of each HP-SEC allergoid fraction (F1-F4) was compared to the protein profile and immunoblot of the native mix grass (GE) and rye grass (RE) extracts. Protein band corresponding to group 1 homologue was detected at 30 kDa in native preparations. Allergoid fractions demonstrated a shift in molecular weight distribution towards a higher Mw range and absence of a protein band corresponding to the Lol p1

The fractions exhibited a decrease in molecular weight in line with increased retention time from 10.34 min to 17.22 min (Additional file 2: Figure S2). The allergen content of each fraction was confirmed by LC-MS/MS analysis (Table 2) and SDS-PAGE/Western blotting (Fig. 1). All fractions contained a group 1 allergen homologue.

**Table 2:** Allergen repertoire identified by LC-MS/MS analysis from the mix grass allergoid HP-SEC fractions. All fractions contained a group 1 allergen homologue.

Grass species	Allergen	Accession number	Fraction 1 (2,249,594 Da)		Fraction 2 (973,404 Da)		Fraction 3 (424,493 Da)		Fraction 4 (113,736 Da)	
			Sequence coverage	Peptide number	Sequence coverage	Peptide number	Sequence coverage	Peptides number	Sequence coverage	Peptide number
*Agrostis capillaris* (Bent grass)	Agr a 1	gi|320606	-	-	-	-	46%	2	46%	2
	Dac g 2	gi|1093120	-	-	-	-	17%	4	22%	5
*Dactylis glomerata* (Orchard grass/Cocksfoot)	Dac g 3	gi|14423759	14%	2	-	-	-	-	46%	4
	Dac g 5	gi|14423122	-	-	-	-	31%	6	31%	8
*Holecus lanatus* (Velvet grass/Yorkshire fog)	Hol l 1	gi|3860384	-	-	-	-	28%	8	37%	9
	Hol l 5	gi|2266625	5%	2	9%	2	21%	4	34%	8
*Lolium perenne* (Perennial ryegrass)	Lol p 1	P14946	12%	4	-	-	25%	7	30%	8
	Lol p 2a	gi|126386	-	-	-	-	36%	4	57%	6
	Lol p 3	P14948	14%	2	-	-	34%	4	34%	4
	Lol p 4	gi|55859464	30%	11	30%	14	36%	16	37%	15
	Lol p 5a	gi|332278195	4%	2	12%	3	24%	6	26%	5
	Lol p 5b	gi|249 8 582	-	-	7%	2	12%	3	27%	7
	Lol p 11	Q7M1X5	19%	3	23%	4	41%	13	13%	12
*Phleum protense* (Timothy grass)	Phl p 1	gi|1582250	15%	4	22%	6	20%	6	30%	8
	Phl p 2	gi|1171009	-	-	-	-	-	-	19%	2
	Phl p 3	gi|39841264	-	-	-	-	39%	3	39%	3
	Phl p 4	gi|54144334	31%	14	32%	16	37%	19	23%	11
	Phl p 5/5 a/5 b	gi|474100420	4%	2	-	-	24%	6	26%	7
	Phl p 6	P43215	25%	2	-	-	45%	6	46%	7
	Phl p 7	gi|14423846	-	-	-	-	29%	2	29%	2
	Phlp p 11	gi|47606039	-	-	30%	5	51%	10	50%	8
*Poa pratensis* (Kentucky bluegrass)	Poa a 1	gi|320620	-	-	-	-	-	-	46%	2

SDS-PAGE and Western blotting were used to compare changes in protein profile and group 1 (Lol p 1-specific) IgG reactivity for HP-SEC fractions from the mix grass modified preparation versus the mix grass native extract. Protein aggregation following glutaraldehyde modification is typically seen as a high molecular weight smear without distinctive banding. A molecular weight distribution pattern towards higher ranges was evident for all four allergoid fractions (Fig. 1a). A decrease in molecular weight of HP-SEC fractions from 2,249,594 Da to 113,736 Da corresponded to a gradual reduction in molecular weight signal across the protein profile. The native mix grass extract exhibited a typical allergen profile of well-defined protein bands between 5 kDa and 250 kDa. A prominent protein band corresponding to the group 1 allergen homologue was detected at 30 kDa in the mix grass native extract. This band was not detected in HP-SEC allergoid fractions confirming modification of group 1 allergen homologue. Retention of Lol p 1 specific IgG reactivity within modified fractions was also confirmed by immunoblotting with anti-Lol p 1 immunodominant polyclonal IgG sera (Fig. 1b).

### Grass specific IgE and IgG reactivity of a native and modified mix grass extracts

**Fig. 2:**
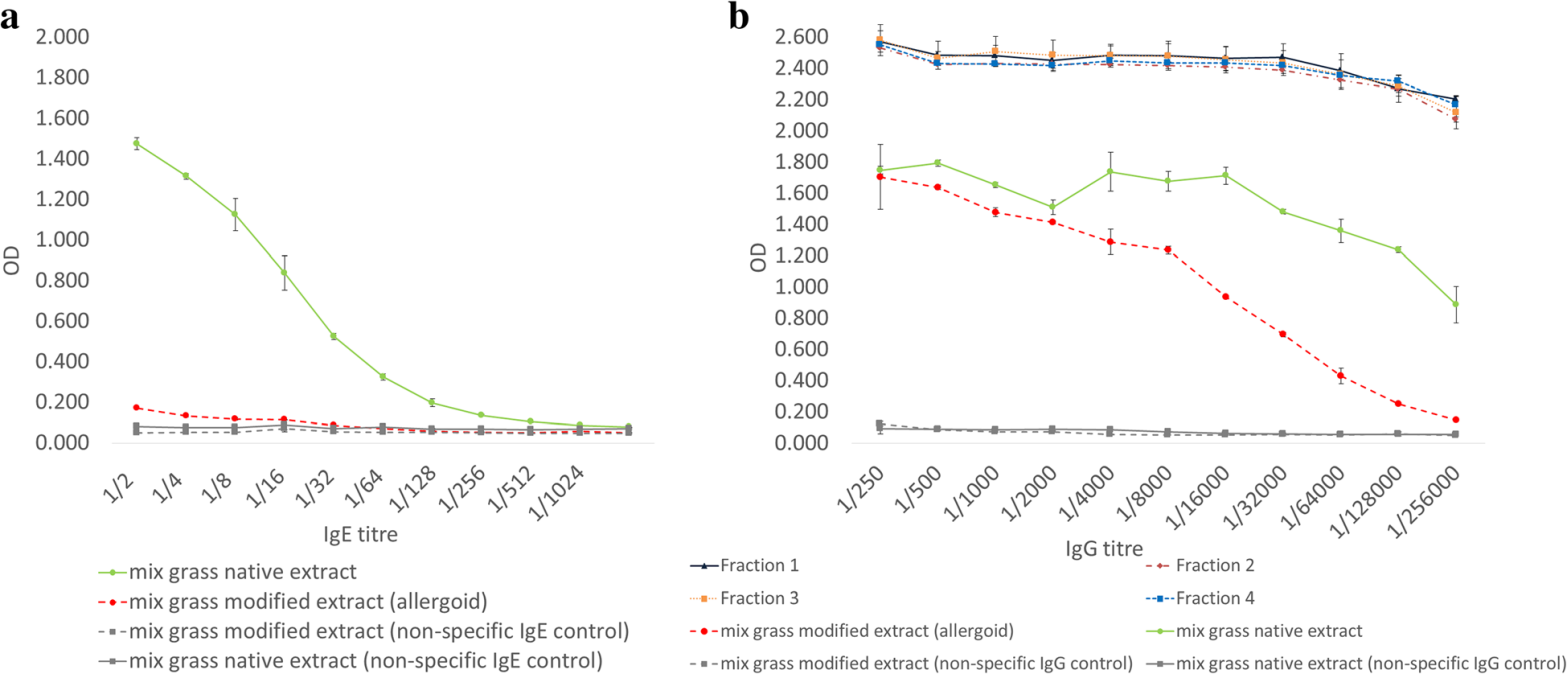
IgE/IgG dose responses of a native and modified mix grass extracts. **a** Grass specific IgE reactivity of the intact native and modified mix grass extracts. **b** Anti-Lol p 1 specific IgG reactivity of HP-SEC fractions from the mix grass modified extract. Specific Lol p 1 IgG response from each fraction was measured using direct ELISA. The data are displayed as mean of duplicate samples +/− standard deviation

Grass specific IgE reactivity and thus the presence of IgE-binding epitopes in native and modified mix grass extracts was determined using direct ELISA technology with human grass specific IgE sera. A typical dose response curve for native mix grass extract can be seen in Fig. 2a. An observed reduction in IgE reactivity from the modified mix grass extract provides evidence of diminished IgE epitope reactivity following modification process (Fig. 2a).

A difference in IgG profile is observed between the whole native extract versus whole allergoid extract (Fig. 2b). Individual HP-SEC size fractions of the allergoid formulation verified maintenance of IgG reactive epitopes across separated size distributions of the allergoid formulation.

### Mapping of group 1, Lol p 1 specific, IgG-binding epitopes and their functional capacities within the mix grass allergoid formulation

**Fig. 3:**
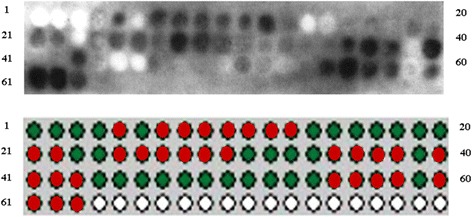
Lol p 1 synthetic peptide sequences recognized by anti-Lol p 1 immunodominant polyclonal rabbit IgG. *Red dots* indicate individual overlapping synthetic peptides positive for anti-Lol p 1 immunodominant polyclonal rabbit IgG binding

Sixty-three synthetic peptides (Additional file 3: Figure S3) with 11 overlapping residues were synthesized to cover the amino acid sequence of the group 1 allergen, Lol p 1, and applied to a cellulose membrane. The membrane was probed with anti-Lol p 1 immunodominant polyclonal rabbit IgG sera. Positive spots, indicative of anti-Lol p 1 IgG binding, were detected for a number of overlapping peptides (Fig. 3).

**Fig. 4:**
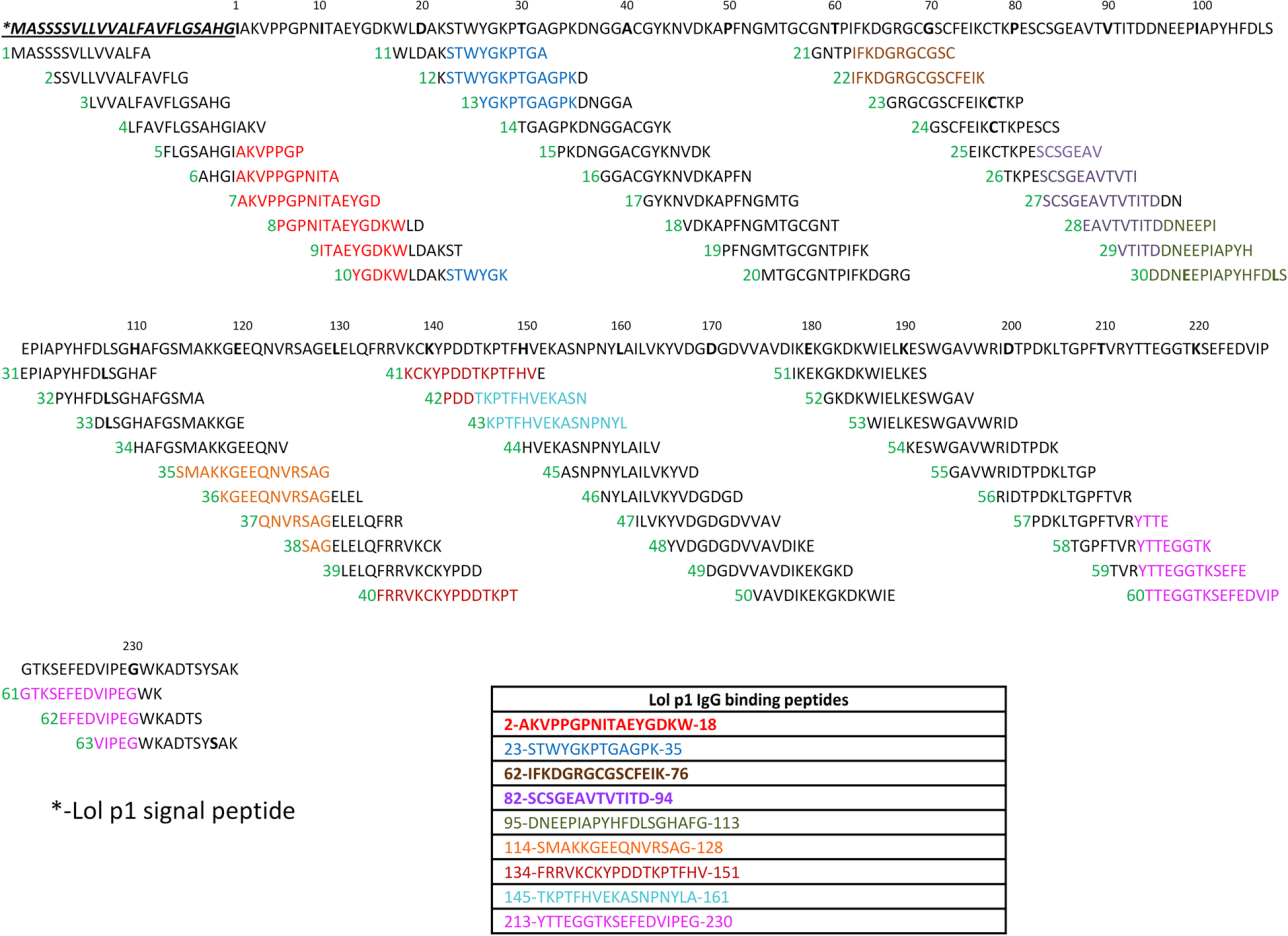
Position of each individual overlapping synthetic peptide on the ORF of Lol p 1 sequence. Positive anti-Lol p 1-binding peptide sequences are highlighted in colour. Nine synthetic peptides were identified as anti-Lol p 1 IgG binding sequences

The position of anti-Lol p 1 IgG-binding peptide sequences on the open reading frame (ORF) of the Lol p 1 amino acid sequence is indicated in the Fig. 4. Overall, nine Lol p 1 specific peptides were identified as being specific for binding anti-Lol p 1 immunodominant polyclonal IgG sera (Fig. 4).

Serial dilutions of the mix grass intact allergoid formulation were used to test the capacity of Lol p 1-specific allergoid epitopes to inhibit IgG binding to plate-bound Lol p 1 synthetic peptides identified from the screening experiments (Fig. 4). Inhibitory capacity of the mix grass modified formulation was exhibited against six out of nine Lol p 1 synthetic peptides (Table 3). The identified Lol p 1 allergoid epitopes displayed various degrees of binding capacity towards anti-Lol p 1 immunodominant polyclonal rabbit IgG (Table 3).

**Table 3:** Lol p 1 IgG-binding epitopes specificity (% of IgG binding) of the mix grass allergoid formulation

		Allergoid concentration (μg/mL)				
		264.0	64.0	16.0	4.0	1.0
Peptide 1	23-STWYGKPTGAGPK-35^b^	37.3%	24.4%	21.9%	14.0%	6.1%
Peptide 2	62-IFKDGRGCGSCFEIK-76 (novel immunodominant)^a^	79.7%	66.1%	49.2%	30.6%	14.2%
Peptide 3	82-SCSGEAVTVTITD-94 (novel)^a^	6.5%	11.4%	9.8%	2.0%	8.5%
Peptide 4	95-DNEEPIAPYHFDLSGHAFG-113^b^	12.8%	4.8%	-	-	-
Peptide 5	114-SMAKKGEEQNVRSAG-128^b^	36.6%	49.8%	44.0%	21.4%	5.9%
Peptide 6	134-FRRVKCKYPDDTKPTFHV-151^b^	22.6%	4.0%	–	–	–

^a^not previously reported in literature. ^﻿﻿b^Petersen et al. [22]; Esch & Klapper [35]; Tamborini and et al. [23]

**Fig. 5:**
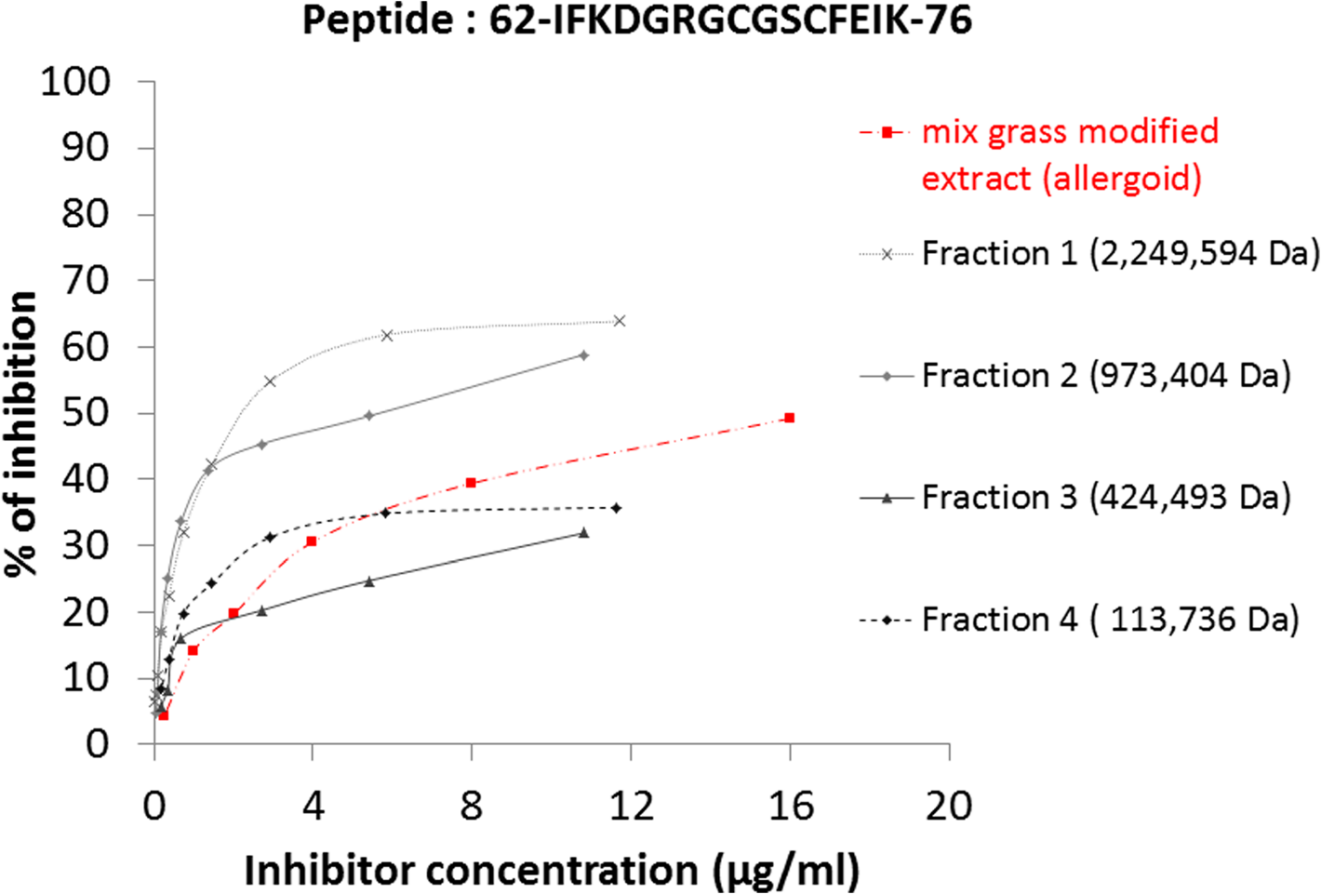
The major Lol p 1 IgG binding epitope from the mix grass allergoid preparation. Mix grass allergoid preparation and isolated HP-SEC allergoid fractions exhibited the highest inhibition for IgG binding against Lol p 1 62-IFKDGRGCGSCFEIK-76 synthetic peptide

HP-SEC fractions from the grass modified formulation with molecular weight ranging between 2,249,594 Da and 113,736 Da were similarly analysed against the immunodominant epitope (62-IFKDGRGCGSCFEIK-76) (Fig. 5). It should be noted that the capacity to inhibit IgG binding to synthetic peptides followed an increasing trend from low to high molecular weight fractions. Screening with the whole allergoid formulation exhibited a profile indicative of an average between the independent fractions.

### Homology model of Lol p 1 IgG-binding epitopes

**Fig. 6:**
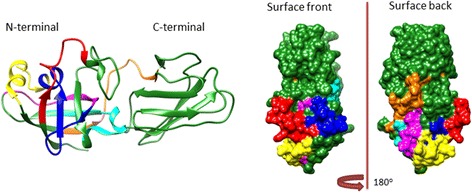
IgG binding epitopes of group 1 grass allergen identified in the mix grass allergoid formulation. The binding regions for Lol p 1 specific IgG are presented as coloured parts of the space filling model (surface front and surface back view) and a ribbon diagram. *Red* (peptide 1); *cyan* (peptide 2); *magenta* (peptide 3); *dark blue* (peptide 4); *yellow* (peptide 5); orange (peptide 6)

A three-dimensional structural model of a group 1 allergen Lol p 1 was built using SWISS-MODEL template library searched with BLAST and HHblits [28]. Primary amino acid sequence of Lol p 1 was obtained from UniProt data base. A template of group 1 allergen from timothy grass Phl p 1 with 89.58% sequence identity to Lol p1 sequence (PBD: 1n10.2A) was used as a template structure to generate the homology model of Lol p 1. IgG binding epitopes from the Table 3 were visualised on the structure (Fig. 6).

All of the identified IgG binding epitopes were localized on the solvent accessible surface of the Lol p 1 structure and accessible to the formation of antibody-antigen complexes. All of the epitopes were located on the N-terminal of Lol p 1 molecule.

## Discussion

The clinical efficacy and safety profile of chemically modified native allergens (allergoids) has been demonstrated in a number of studies [8, 9, 29]. To date, few studies have been presented to characterise allergoid preparations at the molecular level. Glutaraldehyde modification, used in this report, targets free amino groups of amino acid side chains (e.g. lysine) and the reduction in primary amines present in allergoid preparations is used as an indicator of the degree of modification [17]. However, there is limited data published characterising immunogenicity profiles in complex grass allergoid formulations, such as the type of epitopes present and their degree of specificity. Thus, detailed characterisation of antigenic determinants in this type of formulation will help to improve standardisation parameters for allergoid vaccines as well as elucidate aspects associated with their mode of action.

Chemical modification with glutaraldehyde produces stable formulations due to creation of covalent bonds between functional groups. A consistent shift in high molecular weight profile of the allergoid formulation was confirmed in this study with resolution of individual allergoid fractions across different size distributions (Mw 2,249,594-113,736 Da) from HP-SEC. The presence of group 1 homologues within the allergoid formulation was confirmed by LC-MS/MS analysis (Table 2). The overall percentage of modification for the mix grass formulation was 85.51% (data not shown). There are some studies which presented loss or significant reduction in major allergen content for allergoid vaccines [30, 31], which was suggested to result in reduction of clinical and immunological efficacy due to insufficient induction of specific IgG response. Therefore, it is important to demonstrate preservation of clinically relevant major allergens in allergoid preparations and provide assurance that the pharmaceutical development of the formulation has been sufficiently optimised and designed to prevent any loss in active substance. The proteomic approach using HP-SEC and LC-MS/MS analysis was employed to confirm incorporation of the grass allergens in modified preparation (Table 2). Although the allergen forms part of complex polymer chains they still have the capacity to trigger specific immune responses in the form of production of allergen-specific IgG antibody subclasses and, therefore, establishment of immune tolerance against both major and minor cross-reactive grass allergens. While all HP-SEC fractions contained group 1 allergen homologues, there was an increasing trend in the number of identified allergens in fractions of lower molecular weight which is attributed to more efficient tryptic digestion of lower molecular weight portions.

Several literature sources reported that polymerized allergoid vaccines have reduced immunogenicity due to the changes of the protein structure after modification [30, 32], yet still maintain significant IgE allergenic potential which results in therapy-related adverse reactions [33]. However, a lower incidence of systemic reactions with allergoids, as compared to native extracts, in children and adults (two EAACI surveys) has recently been reported [8, 9]. A significant level of reduction for IgE reactivity in the allergoid formulation versus the native extract was demonstrated herein using direct ELISA experiments with grass specific IgE (Fig. 2). Hence, confirming significantly diminished allergic potential of the grass formulation and providing assurance in the quality of the modified preparation. It should be emphasised that previous studies challenging the immunogenicity of allergoids all fail to dissect the preparation across its size distributions. An example of where this exists is noted in the work performed by (but not limited to) Adler et al. [34], in which the authors claim “*reduced histamine release observed for allergoid preparations may be at the expense of immunological efficacy because the chemical modifications lead to a clear reduction in T cell activation and the ability to induce allergen-specific IgG antibody responses*.” The study we present in this report refutes this concept beginning with a logical explanation: An allergoid is not a discreet preparation, but instead a complex set of high molecular weight complexes and in order to reveal its immunogenicity there is a need to unravel its components.

Collecting different size (molecular weight) fractions from the intact mix grass allergoid preparation allows further insights into the IgG-binding capacity of the complexes. This is of particular relevance when considering that after subcutaneous injection the allergoid will be continuously replenished by the interstitial fluid (receiving tissue) and gradually dispersed, thus revealing epitopes that might have been concealed when in a complex with other allergens. As a result, a proportion of epitopes might not be accessible to IgG binding or their accessibility might be significantly reduced due to the heterogeneous complex structure of the allergoid formulation. Therefore, IgG epitope mapping using a whole allergoid preparation provides only limited information about epitopes present and their specificity. Previous studies suggest that polymerization of allergens might induce formation of new and novel epitopes [13]. An increase shift in IgG reactivity was noted between allergoid fractions versus the whole intact preparation and was selected for further investigations to further unravel and provide better resolution of the immunogenicity potential of allergoids.

Immunoscreening using Lol p 1 overlapping synthetic peptides was used to identify linear segments of Lol p 1, which bind to Lol p 1-specific IgG. Four of the six Lol p 1-specific binding sequences (Table 3) which were identified in this study using anti-Lol p 1 immunodominant polyclonal rabbit IgG sera have been reported previously [22, 23, 35]. Peptide 23-STWYGKPTGAGPK-35 corresponds to the IgG binding region on the Lol p 1 structure which has been reported previously by Petersen et al. [22] and Esch & Klapper [35]. Tamborini and co-workers [23] using similar technology of synthetic overlapping peptides mapped three main IgG binding epitopes on Lol p 1, which corresponded to the IgG binding sequences also determined in this study (95-DNEEPIAPYHFDLSGHAFG-113, 114-SMAKKGEEQNVRSAG-128, 134-FRRVKCKYPDDTKPTFHV-151). Two Lol p 1 IgG binding sequences identified in this study (62-IFKDGRGCGSCFEIK-76, 82-SCSGEAVTVTITD-94) have not been previously reported in the literature.

Lol p 1 is one of the most prominent grass allergens to which 95% of grass allergic patients are sensitised [21, 23]. Upon completion of the mapping IgG epitopes, it was important to demonstrate the specificity of retention of Lol p 1 IgG-binding epitopes in the allergoid preparation and determine reveal further structural and immunological insights.

Comparison of IgG binding displacement of synthetic peptides between the intact mix grass allergoid preparation and each of the allergoid fractions demonstrated varying displacement capacity for these epitopes. Furthermore, fractions of larger molecular weight ranges (2,249,594 – 973,404) exhibited increased binding capacity for anti-Lol p 1 IgG in comparison with fractions of lower molecular weight ranges (424,493 – 113,736). The fractionation of the allergoid across its different size (Mw) populations allowed a greater degree of resolution in relation to epitope identification. Therefore, it was important to prove the concept of ‘relative’ epitope specificity for IgG binding from the HP-SEC allergoid fractions in comparison with an intact allergoid formulation.

A major Lol p 1 binding epitope sequence 62-IFKDGRGCGSCFEIK-76 from this study has not been reported as an IgG binding epitope previously. The displacement capacity of up to 80% for this epitope sequence suggests that it is a novel immunodominant Lol p 1 IgG-binding epitope.

A three-dimensional homology model of the Lol p 1 allergen is a central tool for visualization and analysis of IgG binding epitopes. It allows prediction of solvent exposed surfaces, flexibility of the backbone and hydrophilicity of amino acid residues. All these parameters play a major role in immunogenicity of B cell epitopes [24, 36]. Structural models of the allergens and epitope mapping can be determined using experimental approaches such as crystallography and NMR spectroscopy or computational [24]. Structurally, Lol p 1 consists from two major domains: double-psi β-barrel (DPBB) and domain-2 which has an Ig-like fold [36]. There is no data presented regarding which of the two domains is predominantly responsible for IgG binding. This study has identified predominant localization of several IgG epitopes at the N-terminal DPBB domain. Furthermore, all the epitopes identified were located on the solvent exposed surface of Lol p 1 structure and therefore, they are accessible to IgG binding.

## Conclusion

- The findings of this study provide a unique insight into the structural and immunological changes which take place following the modification process of a complex grass extract.
- Native and modified extracts do not consist of two discrete preparations but are a complex heterogeneous formula of native and modified allergen sizes and structures, respectively.
- As a consequence, different sized proportions of the grass formulation revealed distinct immunological profiles and repertoires of IgG binding epitopes not previously reported.
- Structural considerations of complex polymerised allergoid formulations are important to help further characterise their immunological properties.
- The results from this study support the concept that modification allows shorter-course therapy options as a result of providing an IgG epitope repertoire important for efficacy. Additionally, the work paves the way to help further develop methods for standardising allergoid platforms.

## Additional files

Additional file 1: Figure S1. HP-SEC chromatograms of the mix grass allergoid. (TIF 87 kb)

Additional file 2: Figure S2. Lol p 1 synthetic peptide. (TIF 260 kb)

Additional file 3: Figure S3. Individual HP-SEC chromatograms of the grass allergoid fractions. (TIF 43 kb)

## Abbreviations

AA: Amino acid
BSA: Bovine serum albumin
DPBS: Dulbecco phosphate buffered saline
DTT: 1,4 – Dithiothreitol
ELISA: Enzyme-linked immunosorbent assay
HPLC: High performance liquid chromatography
HP-SEC: High performance size exclusion chromatography
HRP: Horse radish peroxidase
IgE: Immunoglobulin E
IgG: Immunoglobulin G
kDa: Kilo Dalton
LC-MS/MS: Liquid chromatography-mass spectrometry
Mw: Molecular weight
OD: Optical density
R_S_: Peak resolution
RT: Retention time
SCIT: Subcutaneous immunotherapy
SDS-PAGE: Sodium dodecyl sulfate polyacrylamide gel electrophoresis
TBS: TRIS buffered saline
TGFβ: Transforming growth factor β
Th: T helper cell
TLR: Toll-like receptor
TMB: 3,3’,5,5’-tetramethylbenzidine peroxidase substrate
WFI: Water for injection
