# Supplementary figures and images for: Molecular fingerprinting of complex grass allergoids: size assessments reveal new insights in epitope repertoires and functional capacities
Source: World Allergy Organ J. 2017 Apr 24;10(1):17. doi: 10.1186/s40413-017-0146-3 (PMC5402054; doi:10.1186/s40413-017-0146-3)

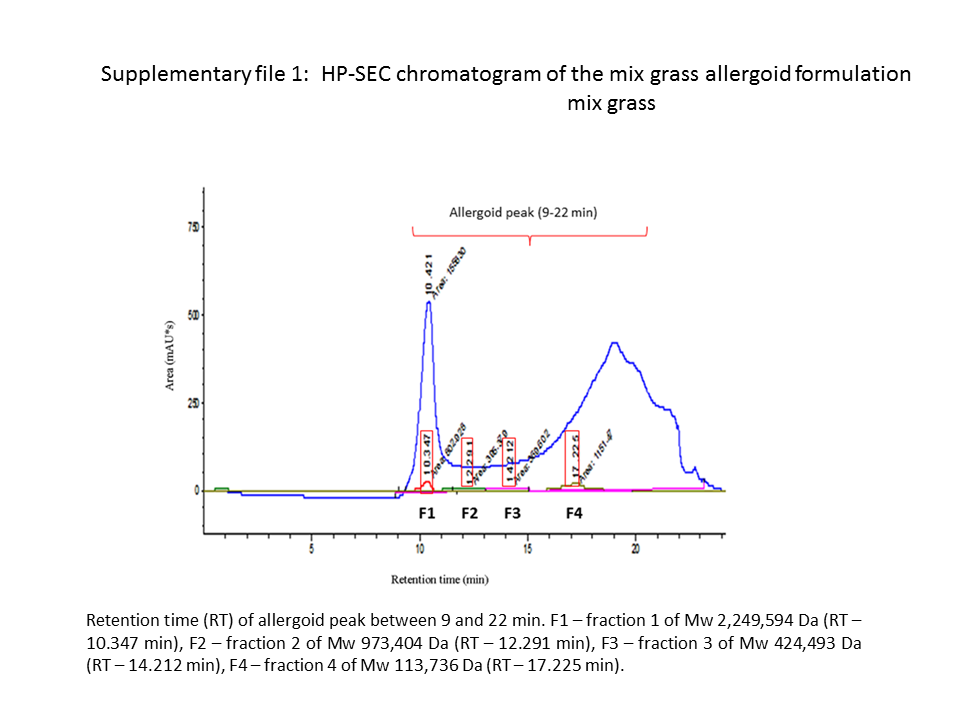

Supplement: Supplementary file 1 — HP-SEC chromatograms of the mix grass allergoid. (TIF 87 kb) [file 40413_2017_146_MOESM1_ESM.tif]

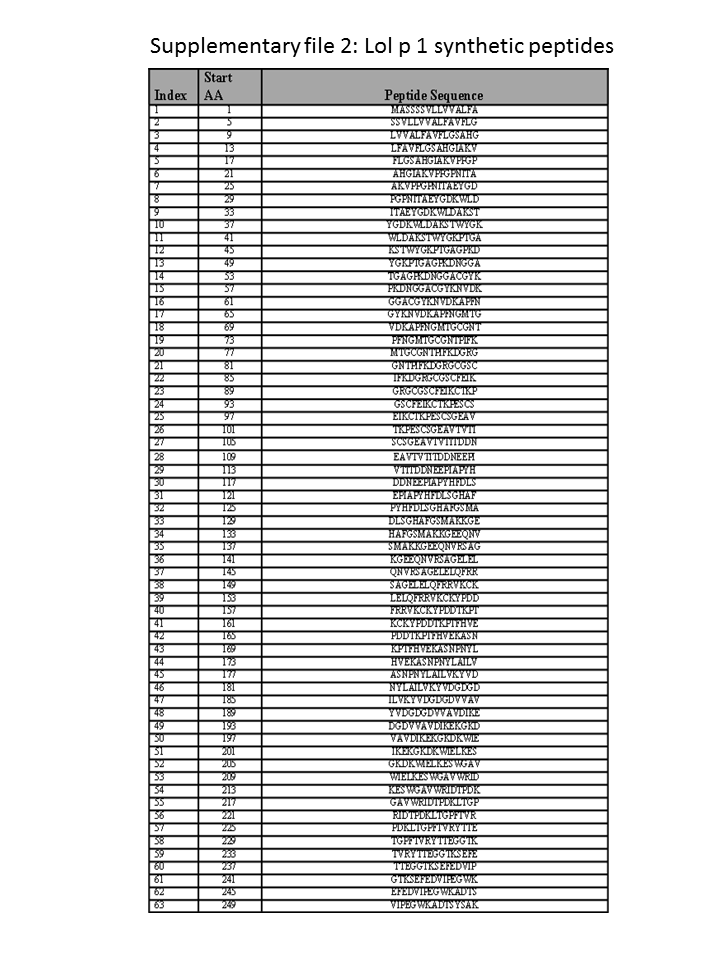

Supplement: Supplementary file 2 — Lol p 1 synthetic peptide. (TIF 260 kb) [file 40413_2017_146_MOESM2_ESM.tif]

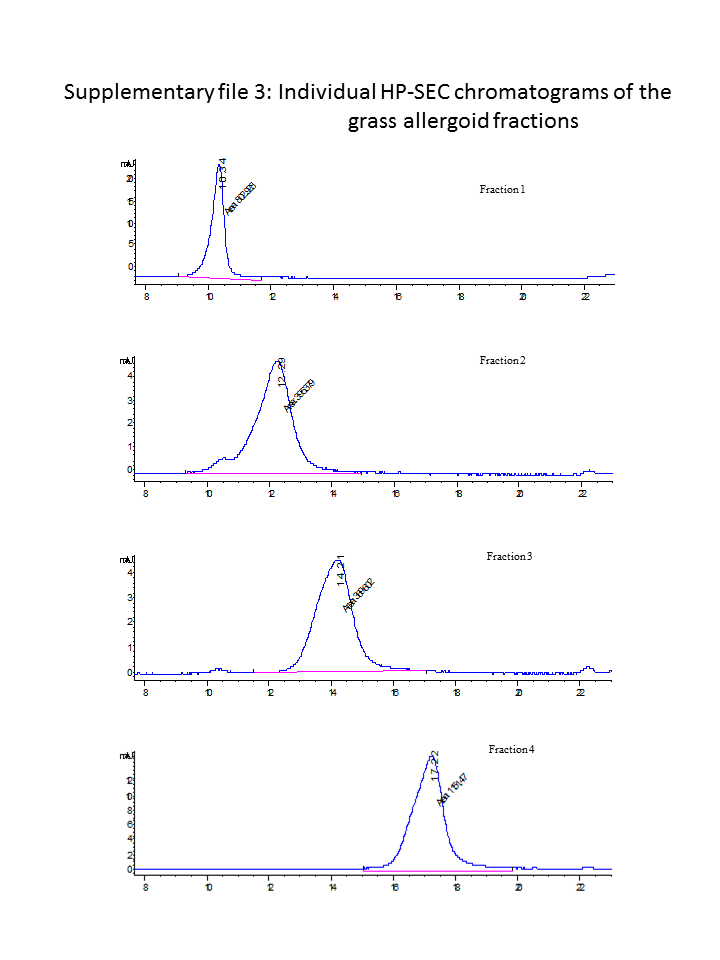

Supplement: Supplementary file 3 — Individual HP-SEC chromatograms of the grass allergoid fractions. (TIF 43 kb) [file 40413_2017_146_MOESM3_ESM.tif]
